# Innate and adaptive stimulation of murine diverse NKT cells result in distinct cellular responses

**DOI:** 10.1002/eji.201847647

**Published:** 2018-12-03

**Authors:** Prabhanshu Tripathi, Saikiran K. Sedimbi, Avadhesh Kumar Singh, Linda Löfbom, Shohreh Issazadeh‐Navikas, Siegfried Weiss, Irmgard Förster, Mikael C. I. Karlsson, Ulf Yrlid, Nadir Kadri, Susanna L. Cardell

**Affiliations:** ^1^ Department of Microbiology and Immunology Institute of Biomedicine University of Gothenburg Gothenburg Sweden; ^2^ Department of Microbiology Tumor and Cell Biology Karolinska Institutet Stockholm Sweden; ^3^ Neuroinflammation Unit Biotech Research and Innovation Centre (BRIC) Faculty of Health and Medical Sciences Copenhagen Biocentre University of Copenhagen Copenhagen Denmark; ^4^ Institute of Immunology Medical School Hannover Hannover Germany; ^5^ Immunology and Environment Life & Medical Sciences (LIMES) Institute University of Bonn Bonn Germany; ^6^ Center of Hematology and Regenerative Medicine Department of Medicine Karolinska Institute Stockholm Sweden

**Keywords:** CD1 molecules, Cytokines, Dendritic cells, NKT cells, Toll like receptors

## Abstract

Natural killer T (NKT) cells recognize glycolipids presented on CD1d. They share features of adaptive T lymphocytes and innate NK cells, and mediate immunoregulatory functions via rapid production of cytokines. Invariant (iNKT) and diverse (dNKT) NKT cell subsets are defined by their TCR. The immunological role of dNKT cells, that do not express the invariant TCRα‐chain used by iNKT cells, is less well explored than that of iNKT cells. Here, we investigated signals driving Toll‐like receptor (TLR) ligand activation of TCR‐transgenic murine dNKT cells. IFN‐γ production by dNKT cells required dendritic cells (DC), cell‐to‐cell contact and presence of TLR ligands. TLR‐stimulated DC activated dNKT cells to secrete IFN‐γ in a CD1d‐, CD80/86‐ and type I IFN‐independent manner. In contrast, a requirement for IL‐12p40, and a TLR ligand‐selective dependence on IL‐18 or IL‐15 was observed. TLR ligand/DC stimulation provoked early secretion of pro‐inflammatory cytokines by both CD62L^+^ and CD62L^−^ dNKT cells. However, proliferation was limited. In contrast, TCR/co‐receptor‐mediated activation resulted in proliferation and delayed production of a broader cytokine spectrum preferentially in CD62L^−^ dNKT cells. Thus, innate (TLR ligand/DC) and adaptive (TCR/co‐receptor) stimulation of dNKT cells resulted in distinct cellular responses that may contribute differently to the formation of immune memory.

## Introduction

The initial immune response raised against pathogens is mediated by innate cells that secrete significant amounts of cytokines and promote subsequent adaptive immunity provided by conventional T and B lymphocytes 1. CD1d restricted natural killer T (NKT) cells are innate like T lymphocytes that exhibit properties of both innate and adaptive immune cells. Consequently, they are thought to function as a bridge between these two arms of immunity 2. In response to lipid antigens presented on the non‐polymorphic, major histocompatibility complex class I‐like molecule CD1d, NKT cells can within hours secrete copious amounts of a wide range of cytokines including IFN‐γ, IL‐4 and IL‐17 3. NKT cells have an essential role in the immune response against infectious agents 4. They can be activated by microbial glycolipids presented on CD1d. In addition, they can be activated indirectly by dendritic cells (DC) that have been stimulated by microbes via pattern recognition receptors. Hence activation of NKT cells by a broad range of pathogens can be achieved.

NKT cells harbor characteristics of both T cells and NK cells. They express a TCR and other shared surface markers and produce typical T cell cytokines. They also express several NK receptors and other NK‐associated surface markers 2. NKT cells also share transcription factor expression and dependence with both T cells and NK cells 2, 5, 6, 7. We therefore postulated that NKT cells might have distinct modes of responses to either innate (TCR‐independent) or adaptive (TCR‐dependent) stimulation, corresponding to their NK‐ and T cell‐like characteristics, respectively. Further, it is not clear to what extent TCR‐independent activation of NKT cells via DC requires signals in addition to cytokines. The role of NKT cells in different immune responses is increasingly appreciated, thus, it is of considerable interest to understand the molecular and cellular mechanisms of activation of NKT cells and their response to activation.

NKT cells are classified by their TCR into type 1 or invariant NKT (iNKT) cells if they express the invariant Vα14‐Jα18 TCRα‐chain in the mouse (Vα24‐Jα18 in human) and diverse NKT (dNKT) cells if they express other CD1d‐restricted TCR 8. The latter population is also referred to as type 2 NKT cells. The frequency of iNKT cells is estimated to be higher than dNKT cells in mice, but studies suggest that dNKT cells may be more frequent in humans 9. The current knowledge on NKT cells is almost exclusively derived from studies performed on iNKT cells. This is largely due to their expression of the invariant TCR and the ease in their detection by CD1d‐multimers loaded with the ligand α‐galactosylceramide (αGalCer). iNKT cells have a preactivated/memory phenotype, and respond very rapidly to stimulation through the TCR by secreting diverse cytokines 2. iNKT cells can be activated indirectly through IL‐12 produced by TLR activated DC in combination with weak interactions with endogenous ligands presented on CD1d 10. Moreover, iNKT cells can be activated in the absence of TCR signaling, instead driven by cytokines such as IL‐12 and IL‐18 that induce the production of a limited array of cytokines including IFN‐γ 11, 12, 13. The latter situation can arise when DC are activated by microbes lacking iNKT cell lipid ligands or microbial products to secrete cytokines, which leads to the activation of iNKT cells.

dNKT cells are less well studied than iNKT cells, mainly due to the lack of specific reagents to identify these cells. However, the available data indicate that dNKT and iNKT cells have distinct functions in specific immune reactions 9. Studies by us and others have demonstrated that dNKT cells can regulate or promote autoimmune disease 14, 15, 16, 17. Further, data suggest that dNKT cells have some features of a resting phenotype (CD62L^+^, CD69^low^, slower cytokine response) compared to iNKT cells, and elicit higher levels of TH1 cytokines such as IFN‐γ rather than TH2 cytokines such as IL‐4 upon activation. Moreover, they recognize distinct ligands 15, 18, 19, 20, 21, 22. Here, we used the 24αβ TCR transgenic mouse model 18 to determine the requirements and cellular interactions leading to the activation of dNKT cells by TLR stimulated DC. Further, we compared the cellular response to this innate mode of activation to the response elicited by adaptive/TCR‐mediated activation.

## Results

### dNKT cell activation by pathogen associated TLR ligands required DC and cell ‐ cell contact

**Figure 1:**
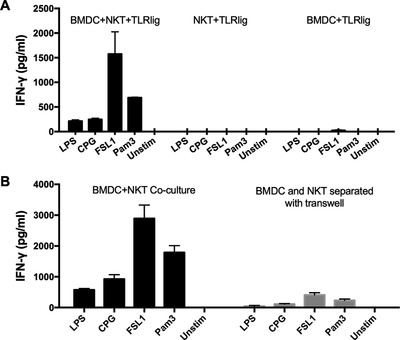
dNKT cell activation by TLR ligands required DC and cell‐to‐cell contact. dNKT cells (NKT) were enriched from spleens of 24αβ transgenic mice, and BMDC were derived from C57BL/6 mice. Cells were cultured as described. Supernatants were harvested at 24 h and IFN‐γ concentration measured by ELISA. (A) NKT cells + BMDC (left), NKT cells (middle) or BMDC (right) were cultured with different TLR ligands (TLR lig). (B) NKT cells and BMDC were cultured in the same chamber (left) or in transwell chambers separated by a membrane (right) in the presence of TLR ligands. Bars indicate mean and SD of triplicate cultures, and show a representative experiment of three (A) and four (B) independent experiments performed.

We found high IFN‐γ production after stimulation of 24αβ TCR transgenic spleen cells with a wide array of TLR ligands that trigger different TLR. The most efficient ligands were LPS that stimulates TLR4, CpG stimulating TLR9, FSL1 that is a synthetic lipopeptide ligand for TLR2/6 and a synthetic lipoprotein agonist, Pam3CSK4, for TLR2/1 (data not shown). We then tested whether 24αβ NKT cells (referred to below as dNKT cells) could be activated directly by TLR ligands, or required the presence of DC 23, 24, as shown before for iNKT cells 10, 12, 25. No IFN‐γ was detected when enriched dNKT cells were stimulated with the TLR ligands in the absence of DC, and TLR stimulation of DC alone yielded no or very low levels of IFN‐γ (only detected after stimulation with FSL1) (Fig. 1A). When stimulating DC, all ligands induced upregulation of CD86 (Supporting Information Fig. 1). However, in the presence of DC, all TLR ligands tested stimulated IFN‐γ production by dNKT cells to varying levels. FSL1 induced the highest amount of IFN‐γ followed by Pam3CSK4. A lower level of IFN‐γ was generally obtained after stimulation with LPS and CpG. In contrast, only very low levels of IFN‐γ were induced when the DC had been pre‐cultured with TLR ligands for 16 h, and washed before co‐culture with NKT cells for 24 h (data not shown). TLR/DC induced IFN‐γ production by dNKT cells was severely reduced in transwell plates separating dNKT cells from DC, in comparison to wells containing co‐cultures of dNKT cells and DC (Fig. 1B). Moreover, conditioned medium from TLR activated DC (50 or 90%) was not able to induce IFN‐γ secretion in dNKT cells (data not shown). Thus, while TLR ligands could not directly activate dNKT cells, dNKT cells were efficiently activated to produce IFN‐γ in the simultaneous presence of ligands and DC. Further, cell‐cell contact between DC and dNKT cells was essential.

### CD1d‐independent TLR/DC activation of dNKT cells

To investigate signals required for TLR/DC induced IFN‐γ production by dNKT cells, we first established that CD5^+^ NK1.1^+^ dNKT cells were major IFN‐γ producing cells after stimulating enriched dNKT cells (Supporting Information Fig. 2A). This enabled us to sort dNKT cells using antibodies to these markers (CD5 and NK1.1) to omit contaminating APC without crosslinking the TCR. Sorted dNKT cells demonstrated the same IFN‐γ responses to TLR/DC stimulation as the enriched cells (Supporting Information Fig. 2B), therefore, sorted dNKT cells (Supporting Information Fig. 3) were used in experiments below where indicated.

**Figure 2:**
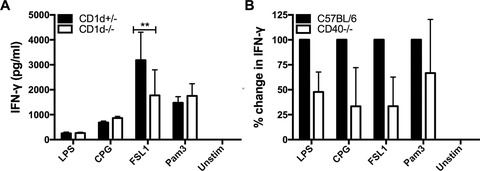
Predominantly CD1d‐independent TLR/DC activation of IFN‐γ production in dNKT cells. dNKT cells (NKT) were sorted from spleens of 24αβ transgenic mice, and BMDC were derived from CD1d^−/−^ and CD1d^+/−^ mice. NKT cells + BMDC were cultured as described. Supernatants were harvested at 24 h and IFN‐γ concentration measured by ELISA. (A) dNKT cells (for sorting strategy, see Supporting Information Fig. 3) were cultured with CD1d^−/−^ or CD1d^+/−^ BMDC in the presence of TLR ligands. NKT cells had been pretreated with CD1d mAb to block CD1d. Bars indicate mean and SD of triplicate cultures, and the results are from one representative experiment of five independent experiments performed. Statistics indicate differences between CD1d^+/−^ and CD1d^−/−^ BMDC for each TLR ligand stimulation using two‐way ANOVA with uncorrected Fisher's LSD post‐hoc test (**p<0.01). (B) dNKT cells were co‐cultured with CD40^−/−^ or C57BL/6 BMDC in the presence of TLR ligands. The graph shows a compilation of three experiments where the values from cultures with C57BL/6 BMDC were set to 100%, and the bars indicate mean and SD of three experiments.

To identify cell surface interactions that were required for the activation of TLR/DC stimulated dNKT cells, we first determined whether CD1d was necessary. DC from CD1d^−/−^ and CD1d^+/‐^ littermate mice were co‐cultured with dNKT cells in the presence or absence of TLR ligands for 24 h. To rule out the potential involvement of CD1d expressed on the dNKT cells, we treated the dNKT with CD1d antibody prior to culture. Compared to CD1d^+/‐^ DC, DC from CD1d^−/−^ mice stimulated the same levels of IFN‐γ production in dNKT co‐cultures with LPS, CpG and Pam3CSK4. However, there was a consistent but partial reduction of IFN‐γ in cultures where dNKT cells had been stimulated with FSL1 together with CD1d^−/−^ DC (Fig. 2A). Further, TLR/DC stimulation showed a trend toward CD40‐dependence, however, results were somewhat variable and the differences compared to cultures with WT DC did not reach significance in our set of experiments (Fig. 2B). In contrast, although the TLR ligands strongly induced CD86 expression on DC, interactions with CD80/86 on DC had no effect on TLR/DC stimulated IFN‐γ production by dNKT cells (Supporting Information Fig. 4A).

### Dependence on IL‐12 for TLR/DC induced cytokine production in dNKT cells

**Figure 3:**
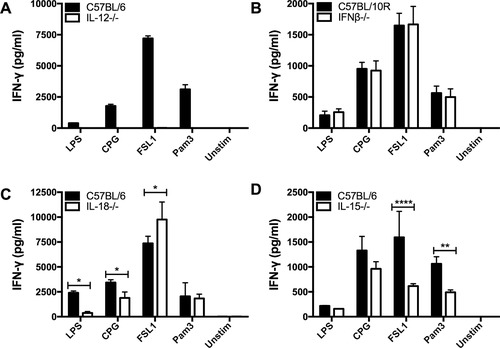
A requirement for IL‐12, and TLR‐specific dependence on IL‐15 and IL‐18 for TLR/DC induced IFN‐γ production by dNKT cells. dNKT cells (NKT) were sorted from spleens of 24αβ transgenic mice, and BMDC were derived from cytokine deficient and C57BL control mice as indicated. NKT cells + BMDC were cultured as described. Supernatants were harvested at 24 h and IFN‐γ concentration measured by ELISA. dNKT cells were cultured with (A) IL‐12^−/−^ (IL‐12p40^−/−^), (B) IFN‐β^−/−^, (C) IL‐18^−/−^ and (D) IL‐15^−/−^ BMDC, or control BMDC, in the presence of TLR ligands. (A–D) Bars indicate mean and SD of triplicate cultures, and show a representative experiment of at least three independent experiments performed for each panel. Statistics indicate differences between cytokine deficient and control BMDC for each TLR ligand stimulation using two‐way ANOVA with uncorrected Fisher's LSD post‐hoc test (*p<0.05, **p<0.01, ****p<0.0001). IFN‐γ was undetectable in cultures with TLR ligands and BMDC from IL‐12^−/−^ (IL‐12p40^−/−^) mice (A).

IL‐12 and IL‐18 have been shown to play a role in TLR/DC induced IFN‐γ production by iNKT cells. Production of IFN‐γ was induced in absence of TCR stimulation when these cytokines were added to purified iNKT cells 12. Here we found that DC deficient in IL‐12p40 were unable to induce IFN‐γ production in dNKT cells in presence of TLR ligands (Fig. 3A). IL‐12p40 is also a component of IL‐23, together with the IL‐23 p19 subunit. DC deficiency in IL‐23p19 resulted instead in an increased IFN‐γ production ruling out a dependence of IL‐23 (Supporting Information Fig. 4B). The increased IFN‐γ production may be due to excess of IL‐12p40 in the absence of the IL‐23p19, and enhanced secretion of IL‐12 by DC 26. Together this demonstrates an absolute IL‐12 dependence of TLR/DC stimulated IFN‐γ production by dNKT cells.

IFN‐β was previously shown to enhance the production of IFN‐γ by iNKT cells induced by DC and TLR9, while IL‐12 was not required 25. In contrast, we did not observe a significant reduction in IFN‐γ secretion by dNKT cells induced by IFN‐β deficient DC together with any of the TLR ligands, including CpG (TLR9 ligand) (Fig. 3B).

### TLR ligand specific dependence on IL‐15 and IL‐18

Further analysis of the role of DC derived cytokines demonstrated a differential dependence on IL‐18 for the induction of IFN‐γ in dNKT cells. IFN‐γ induced by DC and LPS or CpG showed a prominent dependence on IL‐18, while DC and FSL1 or Pam3CSK4 induced IFN‐γ production independently of IL‐18 (Fig. 3C). Previous studies have demonstrated the importance for IL‐15 for the development and maintenance of NK cells, and similarly, homeostasis of iNKT cells depends on IL‐15 27, 28, 29, 30, 31. We found that IL‐15 played a role in TLR/DC activation of dNKT cells specifically by FSL1 and Pam3CSK4, but not a role significant in activation by LPS and CpG (Fig. 3D). Thus, we demonstrate that induction of IFN‐γ production by dNKT cells by TLR ligands + DC has an absolute dependence on IL‐12, and a TLR ligand specific dependence on IL‐18 (LPS and CpG) and IL‐15 (FSL1 and Pan3CSK4).

### IL‐15, IL‐12 and IL‐18 were sufficient to induce IFN‐γ production in dNKT cells

**Figure 4:**
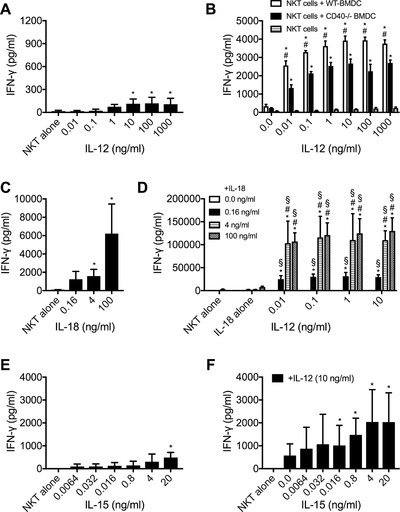
Individual cytokines induced IFN‐γ production in dNKT cells. dNKT cells (NKT) were sorted from spleens of 24αβ transgenic mice (A–F). NKT cells were cultured with the indicated cytokines (A, C–F). (B) NKT cells were cultured with BMDC derived from C57BL/6 mice (WT) or CD40^−/−^ mice as indicated, in the presence or absence of IL‐12. Supernatants were harvested at 24 h and IFN‐γ concentration measured by ELISA. dNKT cells were co‐cultured with specified concentrations of (A) IL‐12 alone, (B) IL‐12 in the presence of C57BL/6 (WT) or CD40^−/−^ BMDC, (C) IL‐18 alone, (D) combinations of IL‐18 and IL‐12, (E) IL‐15 alone and (F) IL‐15 together with IL‐12. Bars indicate mean and SD of three (A, D, E and F) and four (C) independent experiments performed. For (B), a representative experiment is shown of three (dNKT cells + WT‐BMDC and dNKT cells) or two (dNKT cells + CD40^−/−^ BMDC) independent experiments. Statistical comparison are shown for (A) IL‐12 versus dNKT alone (Kruskal–Wallis test); (B) dNKT cells + WT‐BMDC versus dNKT cells (*) and dNKT cells + WT‐BMDC vs. dNKT cells + CD40^−/−^BMDC (#) (ordinary two‐way ANOVA with uncorrected Fisher's LSD post‐hoc test); (C) IL‐18 versus dNKT alone (Kruskal–Wallis test); (D) IL‐18 alone versus the same IL‐18 concentrations combined with different IL‐12 concentrations (*), IL‐12 alone versus the same IL‐12 concentrations combined with different IL‐18 concentrations (§), and different concentrations of IL‐12 combined with 0.16 ng/mL IL‐18 versus IL‐12 combined with 4 or 100 ng/mL of IL‐18 (#) (ordinary two‐way ANOVA with uncorrected Fisher's LSD post‐hoc test); (E) IL‐15 versus dNKT alone (Kruskal–Wallis test); and (F) 10 ng/mL of IL‐12 alone versus IL‐12 combined with IL‐15 (*) (Kruskal–Wallis test). Indicated symbols only show statistical significance (*p*<0.05) and do not show power of significance.

Having demonstrated an important role for cytokines in the TLR/DC mediated induction of IFN‐γ in dNKT cells, we tested whether cytokines alone were able to induce cytokine production. We found that IL‐12 alone could induce some IFN‐γ production, but only to a limited extent regardless of the dose of IL‐12 (Fig. 4A). Interestingly, while 1–10 ng/mL of IL‐12, but not 100 pg/mL, induced IFN‐γ production, the levels detected in co‐cultures with TLR ligands only reached around 20 pg/mL during the 24 hr culture period (Supporting Information Fig. 5A). This suggested that signals other than IL‐12 contributed to the TLR/DC induced cytokine production. Indeed, the addition of unstimulated DC to cultures with dNKT cells and IL‐12 increased IFN‐γ production several‐fold (Fig. 4B and Supporting Information Fig. 5B). This was likely to partly depend on CD40, as CD40‐deficient DC were less efficient. Addition of IL‐18 or IL‐15 alone to dNKT cells also resulted in IFN‐γ production (Fig. 4C, E). IL‐18 plus IL‐12 had a very strong synergistic effect on IFN‐γ secretion (Fig. 4D), however, we found no induction of other cytokines (IL‐10, IL‐17A, TNF, IL‐4, IL‐2) in these cultures (Supporting Information Fig. 5C). In contrast, the addition of IL‐15 together with IL‐12 showed an additive or slightly synergistic effect on IFN‐γ production by dNKT cells (Fig. 4F, cytokines alone in A, E). Thus, we show that in addition to IL‐12 and IL‐18, IL‐15 can contribute substantially to IFN‐γ production in dNKT cells in the absence of TCR and DC derived cell bound signals.

### Adaptive but not innate stimulation induced robust proliferation of dNKT cells

**Figure 5:**
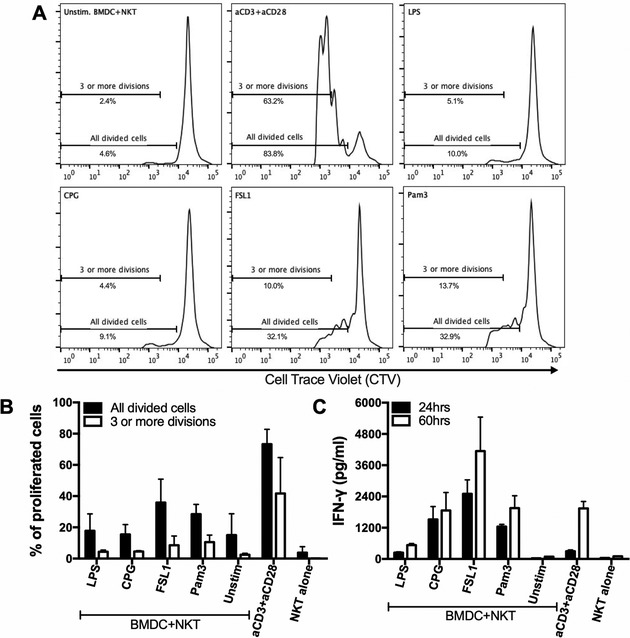
Innate (TLR/DC) activation resulted in rapid cytokine production and low proliferation, whereas adaptive (TCR/CD28) stimulation induced proliferation and late cytokine production. dNKT cells (NKT) were sorted from spleens of 24αβ transgenic mice, and BMDC were derived from C57BL/6 mice. NKT cells were labeled with cell trace violet (CTV) and cultured as described. Supernatants were harvested at 24 and 60 h and IFN‐γ concentration measured by ELISA. Cells were harvested at 60 h and analyzed for CTV dilution (for gating strategy, see Supporting Information Fig. 6). (A, B) CTV labeled sorted dNKT cells were cultured with BMDC in the presence of TLR ligands, or cultured alone with CD3 and CD28 mAbs. (A) Histograms show representative CTV dilution from one representative experiment of three performed, and (B) bars indicate percentage of all divided cells (black bars) and cells with three or more divisions (white bars). (C) IFN‐γ concentrations in supernatants at 24 h (black bars) and 60 h (white bars) of culture was measured by ELISA. Results in (B) and (C) have been compiled, and bars show mean and SD, of three independent experiments.

**Figure 6:**
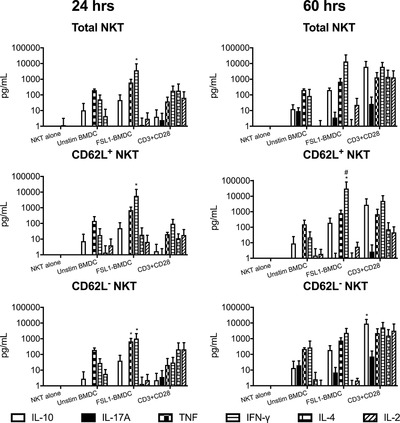
Memory and naive dNKT cells demonstrated distinct response kinetics and cytokine profiles after TLR/DC and TCR/CD28 stimulation. dNKT cells (NKT) were sorted from spleens of 24αβ transgenic mice, and BMDC were derived from C57BL/6 mice. FACS sorted CD62L, CD62L^+^ or total dNKT cells (a mixture of sorted CD62L^+^ and CD62L^−^ dNKT cells) (for sorting strategy, see Supporting Information Fig. 7) were cultured alone with CD3 and CD28 mAbs or in the presence of BMDC and FSL1. Culture supernatants were collected at 24 h and 60 h followed by cytokine analysis using a flow cytometry based Th1/Th2/Th17 cytokine bead array. Supernatants from triplicate wells were pooled for analysis, and bars indicate mean and SD of three independent experiments. Ordinary two‐way ANOVA with uncorrected Fisher's LSD post‐hoc test method was used to calculate statistical significance for FSL1+BMDC versus CD3+CD28 stimulation (*) and CD62L^+^ versus CD62L^−^ (#) for each cytokine. Indicated symbols only show statistical significance (*p*<0.05) and do not show power of significance.

We investigated whether the response of dNKT cells to TLR/DC mediated activation using FSL1 (innate stimulation) differed from the response to TCR mediated activation (adaptive stimulation) by stimulation with antibodies to CD3 and CD28 (TCR/CD28 stimulation). TLR/DC mediated stimulation resulted in a relatively low proliferation, as measured by dilution of cell trace violet (CTV) after 60 h of culture, while TCR/CD28 stimulation induced robust cell division at this time (Fig. 5A, B, and Supporting Information Fig. 6). The difference was especially evident when cells that had divided more than three‐fold were compared. Moreover, the cell recovery was also lower in TLR/DC stimulated cultures, likely due to low cell division and cell death (data not shown). Considering the lower levels of IL‐2 found in TLR/DC compared to TCR/CD28 stimulated cultures (see further below and Fig. 6), we speculated that the low proliferation after TLR/DC stimulation could be due to limiting amounts of IL‐2 in these cultures. Addition of IL‐2 increased cell recovery at 60 h (data not shown), and enhanced the frequency of proliferated cells in TLR/DC stimulated cultures up to two‐fold although the levels observed for TCR/CD28 stimulated cultures were never reached (data not shown). Thus, while TCR/CD28 stimulation induced vigorous proliferation but delayed IFN‐γ secretion, TLR/DC stimulation resulted in rapid and high IFN‐γ production but limited proliferation.

### Distinct responses upon innate and adaptive activation of dNKT cells

We next measured a broad array of cytokines in the TCR/CD28 and FSL1/DC stimulated cultures (Fig. 6 and Supporting Information Fig. 7). At 24 h, IFN‐γ and TNF were more strongly induced by FSL1/DC compared to TCR/CD28 stimulation. In contrast, IL‐17, IL‐4 and IL‐2 levels were consistently higher in TCR/CD28 stimulated cultures. Stimulation with CD3/CD28 mAbs resulted in peak cytokine levels at 60 h after stimulation, while TLR/DC stimulation resulted in robust IFN‐γ, TNF and some IL‐10 production already at 24 h (Fig. 5C and 6). The dNKT cells include cells that are either positive or negative for CD62L that mediates lymph node entry through high endothelial venules, indicating different recirculation patterns and possibly different activation stages. Sorting of dNKT cells into CD62L^+^ or CD62L^−^ fractions before FSL1/DC stimulation demonstrated that IFN‐γ, TNF and IL‐10 was produced at 24 h to comparable levels by dNKT cells with CD62L^+^ or CD62L^−^ phenotype. In the same cultures, IL‐17, IL‐4 and IL‐2 were undetectable or found at low levels. In contrast, after TCR/CD28 stimulation IL‐17, IL‐4 and IL‐2 were produced, primarily by CD62L^−^ dNKT cells with delayed kinetics. Taken together, our data show that TLR/DC stimulation resulted in limited proliferation but early and prominent IFN‐γ and TNF production by both CD62L^+^ and CD62L^−^ dNKT cells. In contrast, TCR/CD28 stimulation resulted in efficient proliferation and delayed secretion of TH1, TH17 and TH2 cytokines preferentially in the CD62L^−^ dNKT subset.

## Discussion

Here we have investigated the requirements for the induction of IFN‐γ production by dNKT cells in response to “innate stimulation” mediated by co‐culture with TLR ligands and DC. We demonstrate that induction of IFN‐γ production required simultaneous presence of TLR ligands and DC in cultures with dNKT cells, and cell‐to‐cell contact was necessary for optimal responses. For all TLR ligands, IFN‐γ induction was strictly dependent on IL‐12 production by DC, while IFN‐β production by DC was dispensable. In contrast, stimulation with TLR ligands varied in their dependence on IL‐18 and IL‐15. The selective cytokine dependence shown by stimulation with the TLR ligands could result from differences in intracellular signaling pathways induced by the different TLR ligands in DC 1. IL‐15 promoted optimal stimulation by FSL1 (TLR2/6) and Pam3CSK4 (TLR1/2). IL‐15 is trans‐presented to NK cells by DC 30, 32, suggesting that trans‐presentation of IL‐15 could contribute to the cell‐to‐cell interaction between DC and dNKT cells. Both TLR2/6 and TLR2/1 activate the MyD88‐dependent intracellular signaling pathway resulting in the activation of NF‐kB and AP‐1 and the expression of pro‐inflammatory cytokines but not type I IFN in DC 1. In contrast, IL‐18 contributed to the response elicited by LPS (TLR4) and CpG (TLR9). TLR9 is expressed in endosomes and activates both the IRF pathway leading to the production of type I IFN and the MyD88 pathway inducing pro‐inflammatory cytokines. Binding of LPS to TLR4 induces its translocation to the endosome and also induces both pro‐inflammatory cytokines through MyD88 and type I IFN through a TRIF/IRF‐dependent pathway. Thus, TLR1/2 and TLR2/6 on the one hand and TLR4 and TLR9 on the other hand share downstream signaling pathways and result in distinct DC responses. Although type I IFN was not required for TLR ligand/DC induced IFN‐γ production by dNKT cells, these TLR signaling pathways may differently induce other contributing accessory signals in DC such as IL‐18 and IL‐15.

We found that IL‐15 alone was sufficient to activate cytokine production, more efficiently than single addition of IL‐12. IL‐15 is important for iNKT cell differentiation and homeostasis 29, 31, but a role in NKT cell activation has to our knowledge not been demonstrated before. The responsiveness to IL‐15 is consistent with the high levels of the IL‐15 receptor component CD122 expressed by the dNKT cells studied here, a level higher than that of iNKT cells 20. It is interesting to note that in vivo priming of NK cell responses, including IFN‐γ production, by TLR ligands (and pathogens) was dependent on DC but was independent on IL‐12 and CD40 30. In contrast, it required the trans‐presentation of IL‐15 by DC to resting NK cells. TLR stimulation of bone marrow derived DC induced the expression of IL‐15Rα, required to trans‐present IL‐15. Moreover, the addition of IL‐15 but not IL‐12 to NK cells in vitro primed NK cells for IFN‐γ production. We found that the effects of IL‐15 and IL‐12 on dNKT cells were essentially additive, suggesting independent effects on dNKT cells, while IL‐12 and IL‐18 provided a strong synergistic induction of IFN‐γ production but not other cytokines tested, consistent with an upregulation of the IL‐18 receptor by IL‐12 on T cells 33.

While stimulation of dNKT cells with DC and LPS, CpG or Pam3CSK4 was CD1d‐independent, we found that stimulation of dNKT cells with FSL1/DC was partially CD1d‐dependent. Stimulation of DC alters lipid metabolism, which can lead to increased display of stimulatory NKT cell lipid ligands on CD1d, as shown for iNKT cells 34, 35, 36, 37, 38, 39. Moreover, the level of CD1d is upregulated on APC upon stimulation with a variety of microbes and TLR ligands 40, 41, 42. FSL1 stimulation of DC might increase the presentation of an agonist lipid ligand/s for 24αβ dNKT cells on CD1d, contributing to dNKT cell stimulation by FSL1/DC. It should be noted that we have used dNKT cells with the 24αβ TCR that recognizes unknown self‐ligands on CD1d, and thus, ligands for other dNKT cell TCR may be regulated differently by DC.

Previous studies have demonstrated that iNKT cells can be activated by TLR ligands and DC 11, 12, 43, 44. One set of studies showed that stimulation of iNKT cells with either LPS or CpG in co‐culture with DC was independent of CD1d, dependent on IL‐12, and optimal stimulation with LPS but not CpG required IL‐18 production by DC 12, 43. In other experiments, a role was described for type I IFN in the innate activation of iNKT cells. CpG was found to activate iNKT cells in a type I IFN and IL‐12 dependent manner, independently of IL‐18 and CD1d 13, 43. Another report that found that CpG induced DC‐production of type I IFN and unknown charged glycosphingolipids, both required for the activation of iNKT cells by CpG 25. We found that both IFN‐β and CD1d were dispensable for TLR/DC (including CpG/DC) activation of IFN‐γ production by dNKT cells. Thus, both iNKT cells and dNKT cells can be induced to produce IFN‐γ by innate stimulation, i.e. by the combination of DC and TLR ligands in the absence of TCR ligation. Our results reinforce the concept that NKT cells can respond to a wide range of pathogens, including viruses, which do not have glycolipid ligands for NKT cells. However, the activation is dependent on DC derived cytokines, of which IL‐12 is most crucial. TCR‐independent innate activation of NKT cells is likely to constitute a physiological in vivo response, as suggested by the lack of detectable TCR signaling in IFN‐γ producing iNKT cells in Nur77‐GFP mice during bacterial and viral infections 44.

T cell ‐ DC interaction via CD40L and CD40 on T cells and DC respectively, increases the production of IL‐12 by DC during TCR stimulation, which in turn increases the production of IFN‐γ by T cells, including iNKT cells 45. Expression of CD40 on DC had a positive effect on dNKT cell cytokine production in response to TLR/DC (Fig. 2B and Fig. 4B). CD40L ligation on dNKT cells did not seem to be important, as in preliminary experiments the addition of plate bound CD40‐Ig or CD40L mAbs did not potentiate IL‐12 induced IFN‐γ production in dNKT cells (data not shown). This suggests that CD40 signaling in DC provided stimulatory factor/s that in turn increased IFN‐γ production in dNKT cells. These factor/s are not likely to be elevated IL‐12 secretion alone, as the addition of increasing concentrations of IL‐12 in combination with CD40‐KO DC could not induce IFN‐γ production to the level obtained with WT DC. Further, the fact that the addition of CD40 deficient DC augmented IL‐12 induced cytokine production suggests that besides CD40, additional signals from DC were necessary for optimal IFN‐γ induction. In contrast, CD80/CD86 did not contribute to TLR/DC stimulation of dNKT cells.

We compared whether adaptive stimulation (TCR/co‐receptor mediated) and innate stimulation (TLR/DC) of dNKT cells lead to different cellular responses. Innate stimulation promoted a relatively rapid cytokine production in dNKT cells regardless of CD62L expression, dominated by pro‐inflammatory cytokines. This response was associated with low cell division. NK cells respond similarly to stimulation by prominent cytokine production without the need for extensive proliferation. Infection with murine cytomegalovirus induces CD1d‐independent IFN‐γ (but not IL‐4) secretion by iNKT cells in the absence of expansion 46, suggesting that in vivo activation of iNKT cells in the absence of TCR signaling may not lead to proliferation. This is consistent with our findings from dNKT cells stimulated in vitro. In contrast, we show that adaptive stimulation resulted in extensive proliferation and a delayed but broader cytokine response primarily by dNKT cells with a CD62L negative phenotype. This response is similar to that of stimulated conventional T cells, in particular to pre‐activated T cells that have an increased capacity to produce a broad range of cytokines including IL‐4 and IL‐10.

Activation by pathogen derived signals and DC in the absence of CD1d ‐ TCR interaction engaged dNKT cells, regardless of activation state, in a robust and rapid production of pro‐inflammatory cytokines. This is likely to have importance for the early response to pathogens, but may not lead to immune memory as little proliferation was induced by this stimulation. However, our data suggest that after stimulation with CD1d‐presented TCR ligands and co‐stimulation, and in the absence of DC‐derived strong pro‐inflammatory cytokines, it is more likely that dNKT cells will respond with secretion of a broader cytokine panel that includes regulatory/TH2 cytokines. Adaptive stimulation of dNKT cells may occur in situations such as in autoimmune disease where self‐lipid agonist ligands may be presented on CD1d and result in the regulation of immunity by dNKT cells through the production of regulatory/TH2 cytokines. The extensive proliferation of dNKT cells occurring in the response to adaptive stimulation suggests that it may also lead to the formation of immune memory. Further, adaptive activation resulted in cytokine production primarily by dNKT cells with a CD62L^−^ phenotype. Interestingly, these cells retained the capacity to respond rapidly to innate stimulation with the secretion of pro‐inflammatory cytokines.

Taken together, our data show that dNKT cells can be activated to produce cytokines both in response to innate stimulation (in the absence of TCR triggering) and following adaptive (TCR/co‐receptor) stimulation. Further, innate and adaptive stimulation of dNKT cells resulted in distinct cellular responses. Innate (TLR/DC) stimulation induced rapid production of pro‐inflammatory cytokines but little proliferation in both naive and memory like dNKT cells. In contrast, adaptive stimulation resulted in robust proliferation, but delayed production of a broader panel of cytokines preferentially by dNKT cells with a pre‐activated phenotype. Thus, our results suggest that the mode of stimulation will have important consequences for dNKT cell functional responses and determine how dNKT contribute to short‐term or memory immune responses.

## Materials and methods

### Mice

C57BL/6 mice were purchased from Charles River, Germany. CD1d^−/−^ 47, IL‐12p40^−/−^ 48, CD40^−/−^ 49, CD80/86^−/−^ 50, IL‐23p19^−/−^ 51 and 24αβ mice 18 backcrossed onto a C57BL/6 genetic background were bred and maintained in a specific pathogen free animal facility at the University of Gothenburg. Sex‐matched male and female mice were used at 8–16 weeks of age. Bone marrow from IL‐15^−/−^ mice 27 (Karolinska Institute, Sweden), and IL‐18^−/−^ mice 52 (University of Bonn, Germany) and C57BL/6 controls were shipped from the indicated universities to the University of Gothenburg and used to derive BMDC in vitro. Bone marrow from IFN‐β^−/−^ mice 53, 54 were shipped from the German Centre for Biotechnology (Braunschweig, Germany) and Copenhagen University (Copenhagen, Denmark) for the same purpose. All experiments were approved by the local animal ethics committee of Gothenburg (approval number 71/16 to S. Cardell).

### NKT cells

Enrichment of NKT cells from 24αβ transgenic spleens was performed by negative selection using CD8, anti‐B220, CD11c and CD11b monoclonal antibodies (mAbs) on an autoMACS Pro Separator (Miltenyi Biotec). The purity was assessed by flow cytometry, and the enriched cells were 87–92% Vα3.2^+^Vβ9^+^ of total live cells. For the majority of experiments, B220^−^CD5^+^NK1.1^+^ cells from 24αβ transgenic spleens were sorted using a BD‐FACS Aria III (for gating strategy for FACS sorting, see Supporting Information Fig. 3). Sorted B220^−^CD5^+^NK1.1^+^ cells were more than 99.5% Vα3.2^+^Vβ9^+^. For some experiments, 24αβ NKT cells were sorted into CD62L^+^ and CD62L^−^ populations using antibodies to CD5, NK1.1 and CD62L (for gating strategy for FACS sorting, see Supporting Information Fig. 7).

### Bone marrow derived dendritic cells (BMDC)

Bone marrow cells were isolated by flushing out mouse femur and tibia, and were matured to DC by in vitro culture with fms‐like tyrosine kinase 3 ligand (FlT3L) in RPMI complete medium (10% fetal bovine serum, 1% penicillin‐streptomycin, 1% HEPES, 1% NaP and 0.1% β‐mercaptoethanol) for 9 days as previously described 23, 24.

### NKT cell stimulation and BMDC co‐cultures

1 × 10^5^ DC were co‐cultured with 1.5 × 10^5^ enriched/sorted 24αβ NKT cells in flat bottom wells in a total volume of 200 μl RPMI complete medium for 24 h in the presence of TLR ligands 10 μg/mL LPS (lipopolysaccharide, ligand for TLR4), 3 μg/mL CpG (CpG oligo dinucleotides, ligand for TLR9), 4 μg/mL FSL1 (synthetic lipoprotein, ligand for TLR6/2) and 4 μg/mL Pam3CSK4 (synthetic bacterial lipopeptide, ligand for TLR1/2) (Invivogen). Transwell culture system (Corning‐Costar) was used to assess the requirement of cell‐cell contact between DC and 24αβ NKT cells. Sorted 24αβ NKT cells were TCR stimulated in CD3 mAb‐coated culture plates (4 μg/mL CD3 mAb over night at 4°C) in the presence of soluble antibody to CD28 (2 μg/mL).

### Flow cytometry

For staining of intracellular cytokines, Brefeldin A (eBiosciences) was added 4 hr before termination of the cultures. Surface staining was performed by first adding Fc block followed by staining with antibodies for Vα3.2 (RR3‐16), Vβ9 (MR10‐2), CD8α (53‐6.7), CD4, (GK 1.5), CD11c (N418) and NK1.1 (PK136) for 30 min on ice. The cells were then fixed and permeabilized using FoxP3 intracellular staining kit (eBiosciences) following manufacturer's instructions. Intracellular staining was performed with either anti‐IFN‐γ (YMG1.2) or isotype control antibodies. Stained cells were acquired on LSRII (BD biosciences) and the data were analyzed using FlowJo software. Guidelines for the use of flow cytometry and cell sorting were adhered to 55.

### Proliferation assay

Cell trace violet (CTV) (Thermo Scientific) labeled 24αβ NKT cells were co‐cultured with BMDC in the presence of the TLR ligands (LPS, CpG, FSL1 or Pam3CSK4) or plate bound CD3 with soluble CD28 mAbs in 96 well plates as described above for 60 h. The cells were harvested after 60 h and analyzed on BD LSR II, total CTV positive cells were considered as 24αβ cells and CTV negative cells as DCs.

### Cytokine detection

Culture supernatants were collected at indicated time points for cytokine analysis using ELISA or cytometric bead array (CBA) kits. IFN‐γ in the cell culture supernatant was measured using an ELISA kit (R&D systems) following the manufacturer's instructions. IFN‐γ, IL‐2, IL‐4, IL‐10, IL‐17 and TNF‐α were measured using the Th1/Th2/Th17 CBA kit (BD biosciences). IL‐12p70 was measured using IL‐12p70 CBA flex sets (BD biosciences).

### Statistics

GraphPad Prism software, version 6 (GraphPad Software, San Diego, CA, USA) was used for all statistical analyses. To compare three or more groups, non‐parametric Kruskal‐Wallis test was used. For analysis of grouped data, two‐way ANOVA with uncorrected Fisher's LSD post‐hoc test or Student's *t*‐test was used to calculate the significance of differences. Data are presented as mean ± SD, and a value of *p* ≤ 0.05 was considered statistically significant.

## Conflict of interest

The authors declare no financial/commercial conflict of interest.

**Abbreviations**
BMDC: bone‐marrow dendritic cells
CTV: cell trace violet
dNKT: NKT cells with diverse TCR
iNKT: NKT cells with invariant TCR
TLR: Toll like receptor

## Supporting information

Peer review correspondence

Supporting information

## References

[1] Takeuchi, O. and Akira, S. , Pattern recognition receptors and inflammation. Cell 2010 140: 805–820. 20303872 10.1016/j.cell.2010.01.022

[2] Bendelac, A. , Savage, P. B. and Teyton, L. , The Biology of NKT Cells. Annu. Rev. Immunol. 2007 25: 297–336. 17150027 10.1146/annurev.immunol.25.022106.141711

[3] Rossjohn, J. , Pellicci, D. G. , Patel, O. , Gapin, L. and Godfrey, D. I. , Recognition of CD1d‐restricted antigens by natural killer T cells. Nat. Rev. Immunol. 2012 12: 845–857. 23154222 10.1038/nri3328 PMC3740582

[4] Tupin, E. , Kinjo, Y. and Kronenberg, M. , The unique role of natural killer T cells in the response to microorganisms. Nat. Rev. Microbiol. 2007 5: 405–417. 17487145 10.1038/nrmicro1657

[5] Godfrey, D. I. and Rossjohn, J. , New ways to turn on NKT cells. J. Exp. Med. 2011 208: 1121–1125. 21646400 10.1084/jem.20110983 PMC3173239

[6] Das, R. , Sant'Angelo, D. B. and Nichols, K. E. , Transcriptional control of invariant NKT cell development. Immunol. Rev. 2010 238: 195–215. 20969594 10.1111/j.1600-065X.2010.00962.x PMC2965566

[7] Cohen, N. R. , Brennan, P. J. , Shay, T. , Watts, G. F. , Brigl, M. , Kang, J. , Brenner, M. B. et al., Shared and distinct transcriptional programs underlie the hybrid nature of iNKT cells. Nat. Immunol. 2013 14: 90–99. 23202270 10.1038/ni.2490 PMC3764492

[8] Godfrey, D. I. , MacDonald, H. R. , Kronenberg, M. , Smyth, M. J. and Van Kaer, L. , NKT cells: what's in a name? Nat. Rev. Immunol. 2004 4: 231–237. 15039760 10.1038/nri1309

[9] Rhost, S. , Sedimbi, S. , Kadri, N. and Cardell, S. L. , Immunomodulatory type II natural killer T lymphocytes in health and disease. Scand. J. Immunol. 2012 76: 246–255. 22724893 10.1111/j.1365-3083.2012.02750.x

[10] Brigl, M. , Bry, L. , Kent, S. C. , Gumperz, J. E. and Brenner, M. B. , Mechanism of CD1d‐restricted natural killer T cell activation during microbial infection. Nat. Immunol. 2003 4: 1230–1237. Epub 2003 Oct 1226. 14578883 10.1038/ni1002

[11] Leite‐De‐Moraes, M. C. , Hameg, A. , Arnould, A. , Machavoine, F. , Koezuka, Y. , Schneider, E. , Herbelin, A. et al., A distinct IL‐18‐induced pathway to fully activate NK T lymphocytes independently from TCR engagement. J. Immunol. 1999 163: 5871–5876. 10570271

[12] Nagarajan, N. A. and Kronenberg, M. , Invariant NKT cells amplify the innate immune response to lipopolysaccharide. J. Immunol. 2007 178: 2706–2713. 17312112 10.4049/jimmunol.178.5.2706

[13] Tyznik, A. J. , Verma, S. , Wang, Q. , Kronenberg, M. and Benedict, C. A. , Distinct requirements for activation of NKT and NK cells during viral infection. J. Immunol. 2014 192: 3676–3685. 24634489 10.4049/jimmunol.1300837 PMC3981072

[14] Jahng, A. , Maricic, I. , Aguilera, C. , Cardell, S. , Halder, R. C. and Kumar, V. , Prevention of autoimmunity by targeting a distinct, noninvariant CD1d‐reactive T cell population reactive to sulfatide. J. Exp. Med. 2004 199: 947–957. 15051763 10.1084/jem.20031389 PMC2211873

[15] Duarte, N. , Stenstrom, M. , Campino, S. , Bergman, M. L. , Lundholm, M. , Holmberg, D. and Cardell, S. L. , Prevention of diabetes in nonobese diabetic mice mediated by CD1d‐restricted nonclassical NKT cells. J. Immunol. 2004 173: 3112–3118. 15322171 10.4049/jimmunol.173.5.3112

[16] Kadri, N. , Korpos, E. , Gupta, S. , Briet, C. , Lofbom, L. , Yagita, H. , Lehuen, A. et al., CD4+ type II NKT cells mediate ICOS and programmed death‐1‐dependent regulation of type 1 diabetes. J. Immunol. 2012 188: 3138–3149. 22371394 10.4049/jimmunol.1101390

[17] Liao, C. M. , Zimmer, M. I. , Shanmuganad, S. , Yu, H. T. , Cardell, S. L. and Wang, C. R. , Dysregulation of CD1d‐restricted type II natural killer T cells leads to spontaneous development of colitis in mice. Gastroenterology 2012 142: 326–334 e321‐322. 22057113 10.1053/j.gastro.2011.10.030 PMC3267843

[18] Sköld, M. , Faizunnessa, N. N. , Wang, C. R. and Cardell, S. , CD1d‐specific NK1.1+ T cells with a transgenic variant TCR. J. Immunol. 2000 165: 168–174. 10861049 10.4049/jimmunol.165.1.168

[19] Stenström, M. , Sköld, M. , Ericsson, A. , Beaudoin, L. , Sidobre, S. , Kronenberg, M. , Lehuen, A. et al., Surface receptors identify mouse NK1.1+ T cell subsets distinguished by function and T cell receptor type. Eur. J. Immunol. 2004 34: 56–65. 14971030 10.1002/eji.200323963

[20] Rolf, J. , Berntman, E. , Stenström, M. , Smith, E. M. K. , Månsson, R. , Stenstad, H. , Yamagata, T. et al., Molecular profiling reveals distinct functional attributes of CD1d‐restricted natural killer (NK) T cell subsets. Mol. Immunol. 2008 45: 2607–2620. 18304639 10.1016/j.molimm.2007.12.022

[21] Chiu, Y. H. , Jayawardena, J. , Weiss, A. , Lee, D. , Park, S. H. , Dautry‐Varsat, A. and Bendelac, A. , Distinct subsets of CD1d‐restricted T cells recognize self‐antigens loaded in different cellular compartments. J. Exp. Med. 1999 189: 103–110. 9874567 10.1084/jem.189.1.103 PMC1887692

[22] Brossay, L. , Tangri, S. , Bix, M. , Cardell, S. , Locksley, R. and Kronenberg, M. , Mouse CD1‐autoreactive T cells have diverse patterns of reactivity to CD1+ targets. J. Immunol. 1998 160: 3681–3688. 9558068

[23] Brasel, K. , De Smedt, T. , Smith, J. L. and Maliszewski, C. R. , Generation of murine dendritic cells from flt3‐ligand‐supplemented bone marrow cultures. Blood 2000 96: 3029–3039. 11049981

[24] Naik, S. H. , Proietto, A. I. , Wilson, N. S. , Dakic, A. , Schnorrer, P. , Fuchsberger, M. , Lahoud, M. H. et al., Cutting edge: generation of splenic CD8+ and CD8‐ dendritic cell equivalents in Fms‐like tyrosine kinase 3 ligand bone marrow cultures. J. Immunol. 2005 174: 6592–6597. 15905497 10.4049/jimmunol.174.11.6592

[25] Paget, C. , Mallevaey, T. , Speak, A. O. , Torres, D. , Fontaine, J. , Sheehan, K. C. , Capron, M. et al., Activation of invariant NKT cells by toll‐like receptor 9‐stimulated dendritic cells requires type I interferon and charged glycosphingolipids. Immunity 2007 27: 597–609. 17950005 10.1016/j.immuni.2007.08.017

[26] Becker, C. , Dornhoff, H. , Neufert, C. , Fantini, M. C. , Wirtz, S. , Huebner, S. , Nikolaev, A. et al., Cutting edge: IL‐23 cross‐regulates IL‐12 production in T cell‐dependent experimental colitis. J. Immunol. 2006 177: 2760–2764. 16920909 10.4049/jimmunol.177.5.2760

[27] Kennedy, M. K. , Glaccum, M. , Brown, S. N. , Butz, E. A. , Viney, J. L. , Embers, M. , Matsuki, N. et al., Reversible defects in natural killer and memory CD8 T cell lineages in interleukin 15‐deficient mice. J. Exp. Med. 2000 191: 771–780. 10704459 10.1084/jem.191.5.771 PMC2195858

[28] Ranson, T. , Vosshenrich, C. A. , Corcuff, E. , Richard, O. , Muller, W. and Di Santo, J. P. , IL‐15 is an essential mediator of peripheral NK‐cell homeostasis. Blood 2003 101: 4887–4893. 12586624 10.1182/blood-2002-11-3392

[29] Ranson, T. , Vosshenrich, C. A. , Corcuff, E. , Richard, O. , Laloux, V. , Lehuen, A. and Di Santo, J. P. , IL‐15 availability conditions homeostasis of peripheral natural killer T cells. Proc Natl Acad Sci U S A 2003 100: 2663–2668. 12598649 10.1073/pnas.0535482100 PMC151397

[30] Lucas, M. , Schachterle, W. , Oberle, K. , Aichele, P. and Diefenbach, A. , Dendritic cells prime natural killer cells by trans‐presenting interleukin 15. Immunity 2007 26: 503–517. 17398124 10.1016/j.immuni.2007.03.006 PMC2084390

[31] Matsuda, J. L. , Gapin, L. , Sidobre, S. , Kieper, W. C. , Tan, J. T. , Ceredig, R. , Surh, C. D. et al., Homeostasis of V alpha 14i NKT cells. Nat. Immunol. 2002 3: 966–974. 12244311 10.1038/ni837

[32] Luu, T. T. , Ganesan, S. , Wagner, A. K. , Sarhan, D. , Meinke, S. , Garbi, N. , Hammerling, G. et al., Independent control of natural killer cell responsiveness and homeostasis at steady‐state by CD11c+ dendritic cells. Sci. Rep. 2016 6: 37996. 27905484 10.1038/srep37996 PMC5131354

[33] Yoshimoto, T. , Takeda, K. , Tanaka, T. , Ohkusu, K. , Kashiwamura, S. , Okamura, H. and Nakanishi, K. , IL‐12 up‐regulates IL‐18 receptor expression on T cells, Th1 cells, and B cells: synergism with IL‐18 for IFN‐g production. J. Immunol. 1999 161: 3400–3407. 9759857

[34] Mallevaey, T. , Zanetta, J. P. , Faveeuw, C. , Fontaine, J. , Maes, E. , Platt, F. , Capron, M. et al., Activation of invariant NKT cells by the helminth parasite schistosoma mansoni. J. Immunol. 2006 176: 2476–2485. 16456008 10.4049/jimmunol.176.4.2476

[35] Paget, C. , Bialecki, E. , Fontaine, J. , Vendeville, C. , Mallevaey, T. , Faveeuw, C. and Trottein, F. , Role of invariant NK T lymphocytes in immune responses to CpG oligodeoxynucleotides. J. Immunol. 2009 182: 1846–1853. 19201836 10.4049/jimmunol.0802492

[36] Darmoise, A. , Teneberg, S. , Bouzonville, L. , Brady, R. O. , Beck, M. , Kaufmann, S. H. and Winau, F. , Lysosomal alpha‐galactosidase controls the generation of self lipid antigens for natural killer T cells. Immunity 2010 33: 216–228. 20727792 10.1016/j.immuni.2010.08.003 PMC4018304

[37] Kain, L. , Webb, B. , Anderson, B. L. , Deng, S. , Holt, M. , Constanzo, A. , Zhao, M. et al., The identification of the endogenous ligands of natural killer T cells reveals the presence of mammalian α‐linked glycosylceramides. Immunity 2014 41: 543–554. 25367571 10.1016/j.immuni.2014.08.017 PMC4220304

[38] Salio, M. , Speak, A. O. , Shepherd, D. , Polzella, P. , Illarionov, P. A. , Veerapen, N. , Besra, G. S. et al., Modulation of human natural killer T cell ligands on TLR‐mediated antigen‐presenting cell activation. PNAS 2007 104: 20490–20495. 18077358 10.1073/pnas.0710145104 PMC2154458

[39] Brennan, P. J. , Tatituri, R. V. , Brigl, M. , Kim, E. Y. , Tuli, A. , Sanderson, J. P. , Gadola, S. D. et al., Invariant natural killer T cells recognize lipid self antigen induced by microbial danger signals. Nat. Immunol. 2011 12: 1202–1211. 22037601 10.1038/ni.2143 PMC3242449

[40] Berntman, E. , Rolf, J. , Johansson, C. , Anderson, P. and Cardell, S. L. , The role of CD1d‐restricted NK T lymphocytes in the immune response to oral infection with Salmonella typhimurium. Eur. J. Immunol. 2005 35: 2100–2109. 15940666 10.1002/eji.200425846

[41] Raghuraman, G. , Geng, Y. and Wang, C. R. , IFN‐beta‐mediated up‐regulation of CD1d in bacteria‐infected APCs. J. Immunol. 2006 177: 7841–7848. 17114455 10.4049/jimmunol.177.11.7841

[42] Skold, M. , Xiong, X. , Illarionov, P. A. , Besra, G. S. and Behar, S. M. , Interplay of cytokines and microbial signals in regulation of CD1d expression and NKT cell activation. J. Immunol. 2005 175: 3584–3593. 16148102 10.4049/jimmunol.175.6.3584

[43] Tyznik, A. J. , Tupin, E. , Nagarajan, N. A. , Her, M. J. , Benedict, C. A. and Kronenberg, M. , Cutting edge: the mechanism of invariant NKT cell responses to viral danger signals. J. Immunol. 2008 181: 4452–4456. 18802047 10.4049/jimmunol.181.7.4452 PMC2597678

[44] Holzapfel, K. L. , Tyznik, A. J. , Kronenberg, M. and Hogquist, K. A. , Antigen‐dependent versus ‐independent activation of invariant NKT cells during infection. J. Immunol. 2014 192: 5490–5498. 24813205 10.4049/jimmunol.1400722 PMC4053538

[45] Kitamura, H. , Iwakabe, K. , Yahata, T. , Nishimura, S. , Ohta, A. , Ohmi, Y. , Sato, M. et al., The natural killer T (NKT) cell ligand alpha‐galactosylceramide demonstrates its immunopotentiating effect by inducing interleukin (IL)‐12 production by dendritic cells and IL‐12 receptor expression on NKT cells. J. Exp. Med. 1999 189: 1121–1128. 10190903 10.1084/jem.189.7.1121 PMC2193012

[46] Wesley, J. D. , Tessmer, M. S. , Chaukos, D. and Brossay, L. , NK cell‐like behavior of Valpha14i NK T cells during MCMV infection. PLoS Pathog. 2008 4: e1000106. 18636102 10.1371/journal.ppat.1000106 PMC2442879

[47] Chen, Y. H. , Chiu, N. M. , Mandal, M. , Wang, N. and Wang, C. R. , Impaired NK1+ T cell development and early IL‐4 production in CD1‐ deficient mice. Immunity 1997 6: 459–467. 9133425 10.1016/s1074-7613(00)80289-7

[48] Magram, J. , Connaughton, S. E. , Warrier, R. R. , Carvajal, D. M. , Wu, C. Y. , Ferrante, J. , Stewart, C. et al., IL‐12‐deficient mice are defective in IFN gamma production and type 1 cytokine responses. Immunity 1996 4: 471–481. 8630732 10.1016/s1074-7613(00)80413-6

[49] Kawabe, T. , Naka, T. , Yoshida, K. , Tanaka, T. , Fujiwara, H. , Suematsu, S. , Yoshida, N. et al., The immune responses in CD40‐deficient mice: impaired immunoglobulin class switching and germinal center formation. Immunity 1994 1: 167–178. 7534202 10.1016/1074-7613(94)90095-7

[50] Borriello, F. , Sethna, M. P. , Boyd, S. D. , Schweitzer, A. N. , Tivol, E. A. , Jacoby, D. , Strom, T. B. et al., B7‐1 and B7‐2 have overlapping, critical roles in immunoglobulin class switching and germinal center formation. Immunity 1997 6: 303–313. 9075931 10.1016/s1074-7613(00)80333-7

[51] Cua, D. J. , Sherlock, J. , Chen, Y. , Murphy, C. A. , Joyce, B. , Seymour, B. , Lucian, L. et al., Interleukin‐23 rather than interleukin‐12 is the critical cytokine for autoimmune inflammation of the brain. Nature 2003 421: 744–748. 12610626 10.1038/nature01355

[52] Hochholzer, P. , Lipford, G. B. , Wagner, H. , Pfeffer, K. and Heeg, K. , Role of interleukin‐18 (IL‐18) during lethal shock: decreased lipopolysaccharide sensitivity but normal superantigen reaction in IL‐18‐deficient mice. Infect. Immun. 2000 68: 3502–3508. 10816504 10.1128/iai.68.6.3502-3508.2000 PMC97635

[53] Erlandsson, L. , Blumenthal, R. , Eloranta, M. L. , Engel, H. , Alm, G. , Weiss, S. and Leanderson, T. , Interferon‐beta is required for interferon‐alpha production in mouse fibroblasts. Curr. Biol. 1998 8: 223–226. 9501984 10.1016/s0960-9822(98)70086-7

[54] Liu, Y. , Carlsson, R. , Comabella, M. , Wang, J. , Kosicki, M. , Carrion, B. , Hasan, M. et al., FoxA1 directs the lineage and immunosuppressive properties of a novel regulatory T cell population in EAE and MS. Nat. Med. 2014: 20: 272–282 1‐13. 24531377 10.1038/nm.3485

[55] Cossarizza, A. , Chang, H. D. , Radbruch, A. , Akdis, M. , Andra, I. , Annunziato, F. , Bacher, P. et al., Guidelines for the use of flow cytometry and cell sorting in immunological studies. Eur. J. Immunol. 2017 47: 1584–1797. 29023707 10.1002/eji.201646632 PMC9165548

