# Supplementary material for: Innate and adaptive stimulation of murine diverse NKT cells result in distinct cellular responses
Source: Eur J Immunol. 2018 Dec 3;49(3):443–53. doi: 10.1002/eji.201847647 (PMC6587840; doi:10.1002/eji.201847647)
Supplement: Supplementary file 2 — Supporting information [file EJI-49-443-s002.pdf]

European Journal of Immunology

Supporting Information
for
DOI 10.1002/eji.201847647

Prabhanshu Tripathi, Saikiran K. Sedimbi, Avadhesh Kumar Singh, Linda Löfbom, Shohreh Issazadeh-Navikas, Siegfried Weiss, Irmgard Förster, Mikael C. I. Karlsson, Ulf Yrlid, Nadir Kadri and Susanna L. Cardell

Innate and adaptive stimulation of murine diverse NKT cells result in distinct cellular responses

© 2018 WILEY-VCH Verlag GmbH & Co. KGaA, Weinheim

Supplemental data

Innate and adaptive stimulation of diverse NKT cells result in distinct cellular responses

Prabhanshu Tripathi[1] *, Saikiran Sedimbi[1,2], Avadhesh Kumar Singh[1], Linda Löfbom[1], Shohreh Issazadeh-Navikas[3], Siegfried Weiss[4], Irmgard Förster[5], Mikael C. I. Karlsson[2], Ulf Yrlid[1], Nadir Kadri[6] and Susanna L. Cardell[1]

[1]Department of Microbiology and Immunology, Institute of Biomedicine, University of Gothenburg, Gothenburg, Sweden

[2] Department of Microbiology, Tumor and Cell Biology, Karolinska Institutet, Stockholm, Sweden

[3] Neuroinflammation Unit, Biotech Research and Innovation Centre (BRIC), Faculty of Health and Medical Sciences, University of Copenhagen, Copenhagen Biocentre, Copenhagen, Denmark

[4] Institute of Immunology, Medical School Hannover, Hannover, Germany

[5] Immunology and Environment, Life & Medical Sciences (LIMES) Institute, University of Bonn, Bonn, Germany

[6] Center of Hematology and Regenerative Medicine, Department of Medicine, Karolinska Institute, Stockholm, Sweden.

Correspondence

Susanna L Cardell
Department of Microbiology and Immunology
Institute of Biomedicine, Box 435
University of Gothenburg, 405 30 Gothenburg, Sweden
susanna.cardell@microbio.gu.se
Phone: +46 31 7866228

A

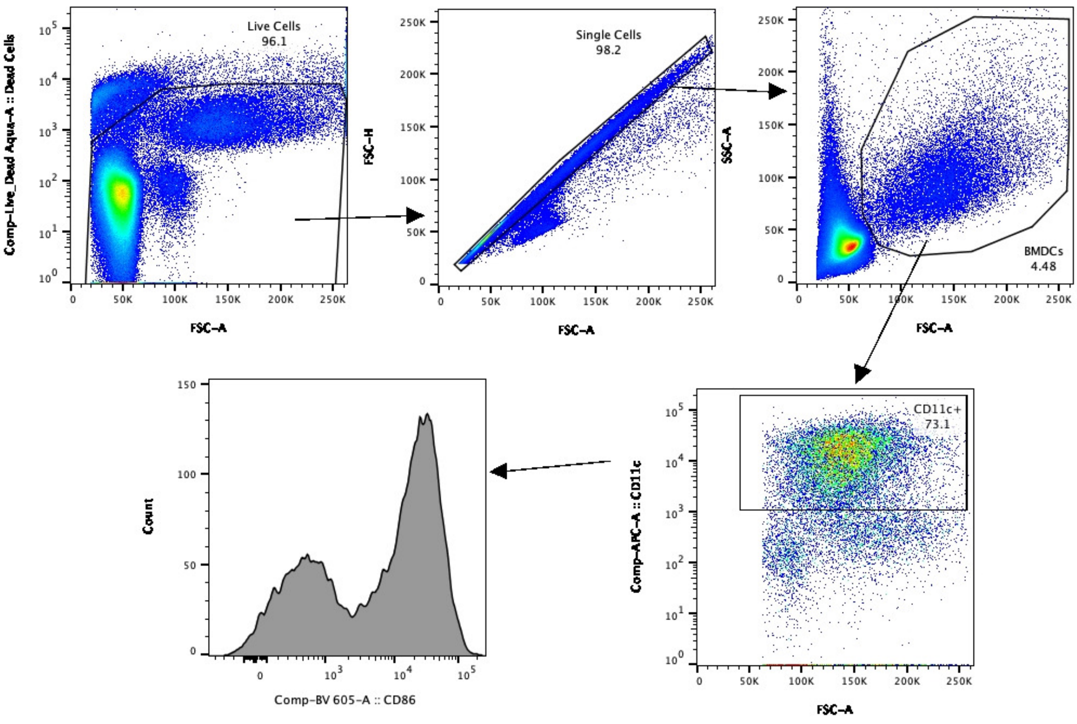

B

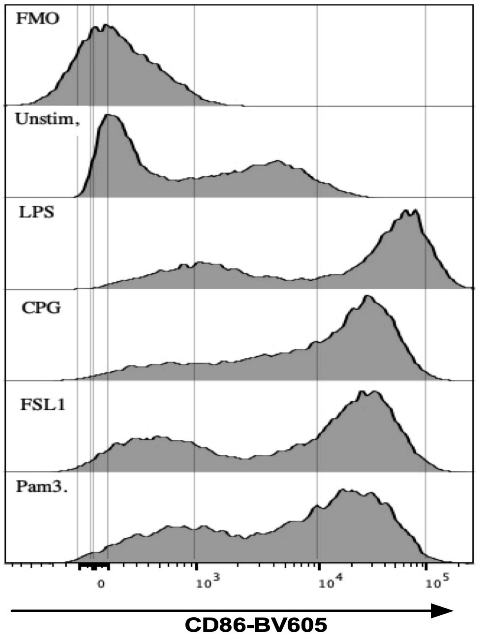

Supplemental Figure 1. *TLR activation of BMDC resulted in increased CD86 expression.* Bone marrow derived dendritic cells (BMDC) were derived from C57BL/6 mice and incubated with TLR ligands for 24 hours. The cells were harvested and stained for flow cytometry analysis as described in Materials and methods. (A) Representative flow cytometry plots for BMDC gating strategy are shown. (B) Histograms show levels of CD86 expression on total CD11c+ BMDC from one representative experiment of two performed.

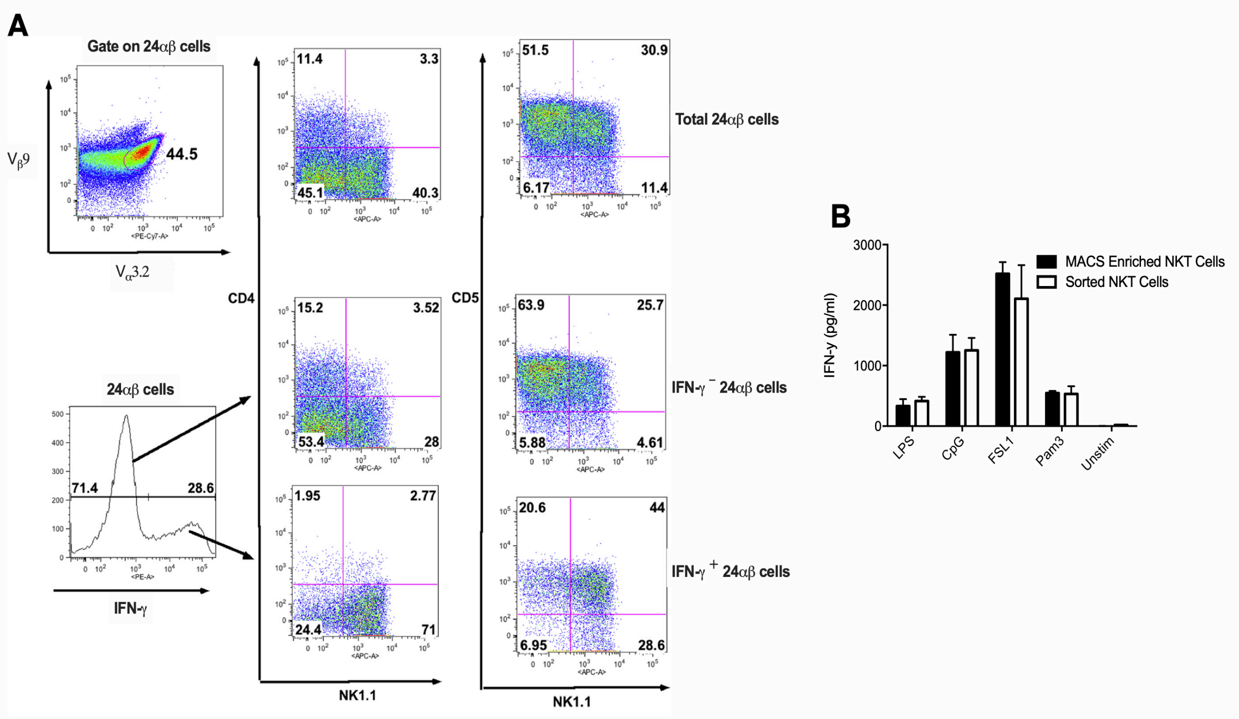

Supplemental Figure 2. *Strategy for sorting of dNKT cells and comparison with MACS enriched dNKT cells for TLR/BMDC induced IFN-γ production*. dNKT cells were MACS enriched (A) or FACS sorted (B) from spleens of 24αβ mice, and BMDC were derived from C57BL/6 mice. (A) Enriched 24αβ dNKT cells were stimulated with BMDC and FSL1. At 24 hours, cells were harvested, stained and analyzed by flow cytometry. 24αβ dNKT cells were gated using antibodies specific for the V-genes of the transgenic TCR (Vβ9 and Vα3.2, upper left panel) and displayed for the expression of CD4 versus NK1. and CD5 versus NK1.1 (upper middle and right panels). The lower left panel shows intracellular IFN-γ staining of FSL1/BMDC stimulated 24αβ dNKT cells gated as above. 24αβ dNKT cells were further gated as indicated into IFN-γ positive or negative cells, and displayed for the expression of CD4 versus NK1.1 (left dotplots) and CD5 versus NK1.1 (right dotplots). B) MACS enriched (black bars) and FACS sorted (white bars) 24αβ dNKT cells were stimulated with BMDC in the presence of different TLR ligands. Supernatant was collected at 24 hours, and IFN-γ measured by ELISA. Bars indicate mean and SD of triplicate cultures. The results shown are from one of two similar experiments.

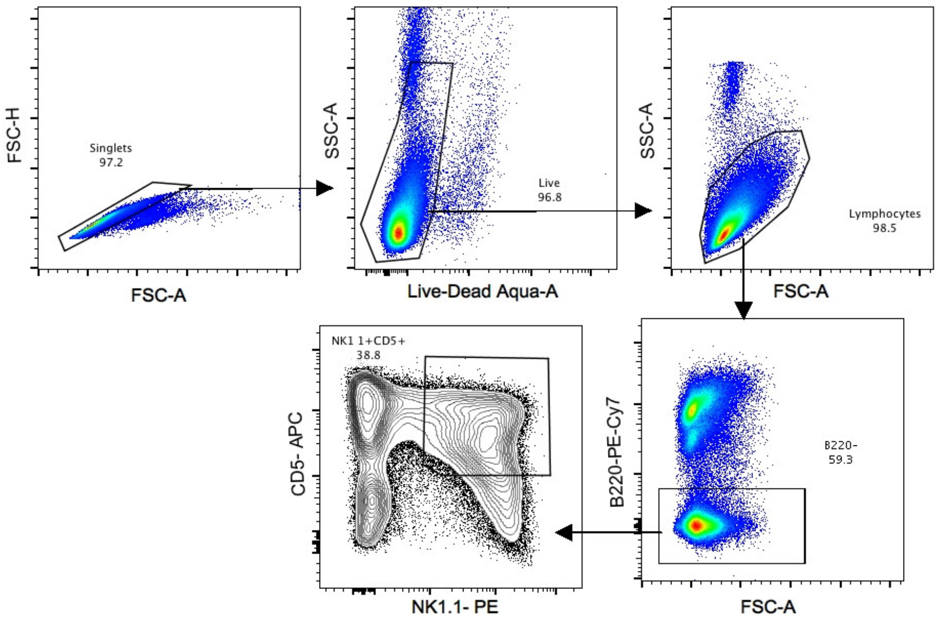

Supplemental Figure 3*. Complete gating strategy for FACS sorting of dNKT cells.* 24αβ dNKT cells were sorted from spleens of 24αβ mice using antibodies to B220, CD5 and NK1.1. $B330^{-}CD5^{+}NK1.1^{+}$ cells gated as shown were sorted and used as sorted pure dNKT cells as indicated in experiments.

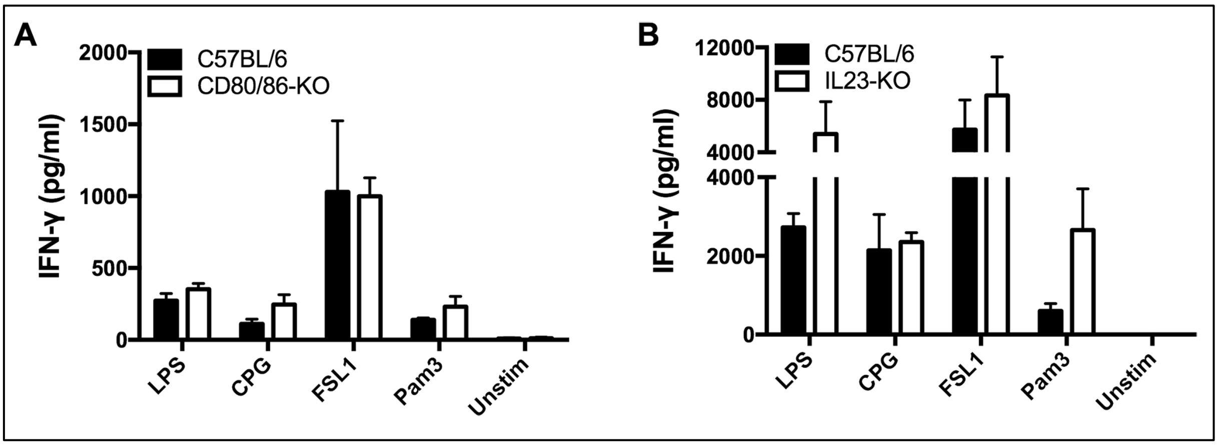

Supplemental Figure 4. *Role of CD80/86 and IL-23p19 expression in BMDC for TLR/BMDC stimulation of dNKT cell IFN-γ production*. dNKT cells were MACS enriched from spleens of 24αβ mice, and BMDC were derived from C57BL/6 mice or the indicated knock-out (KO) mice. Enriched dNKT cells were cultured with BMDC from CD80/86-KO mice (A) or from IL-23p19-KO mice (IL-23-KO) (B) or from C57BL/6 control mice in the presence of different TLR ligands. Supernatants were harvested at 24 hours and IFN-γ measured by ELISA. Bars indicate mean and SD of triplicate cultures, and results are representative of three (A) or two (B) experiments.

A

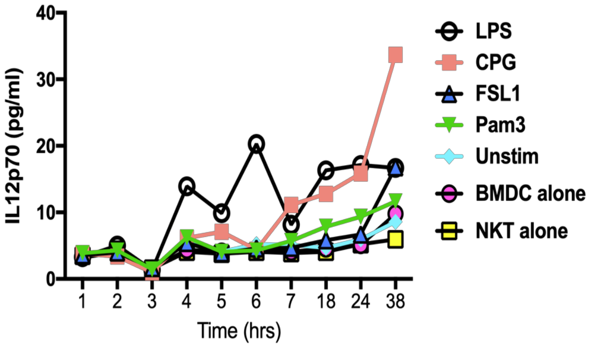
IL12p70 (pg/ml)
40
30
20
10
0
1 2 3 4 5 6 7 18 24 38
Time (hrs)
LPS
CPG
FSL1
Pam3
Unstim
BMDC alone
NKT alone

B

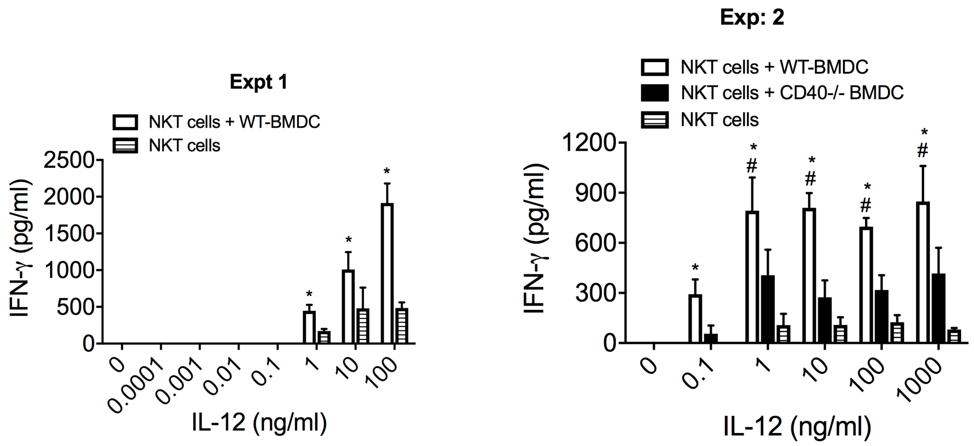
Expt 1
NKT cells + WT-BMDC
NKT cells
IFN-γ (pg/ml)
2500
2000
1500
1000
500
0
0 0.0001 0.001 0.01 0.1 1 10 100
IL-12 (ng/ml)
Exp: 2
NKT cells + WT-BMDC
NKT cells + CD40-/- BMDC
NKT cells
IFN-γ (pg/ml)
1200
900
600
300
0
0 0.1 1 10 100 1000
IL-12 (ng/ml)

C

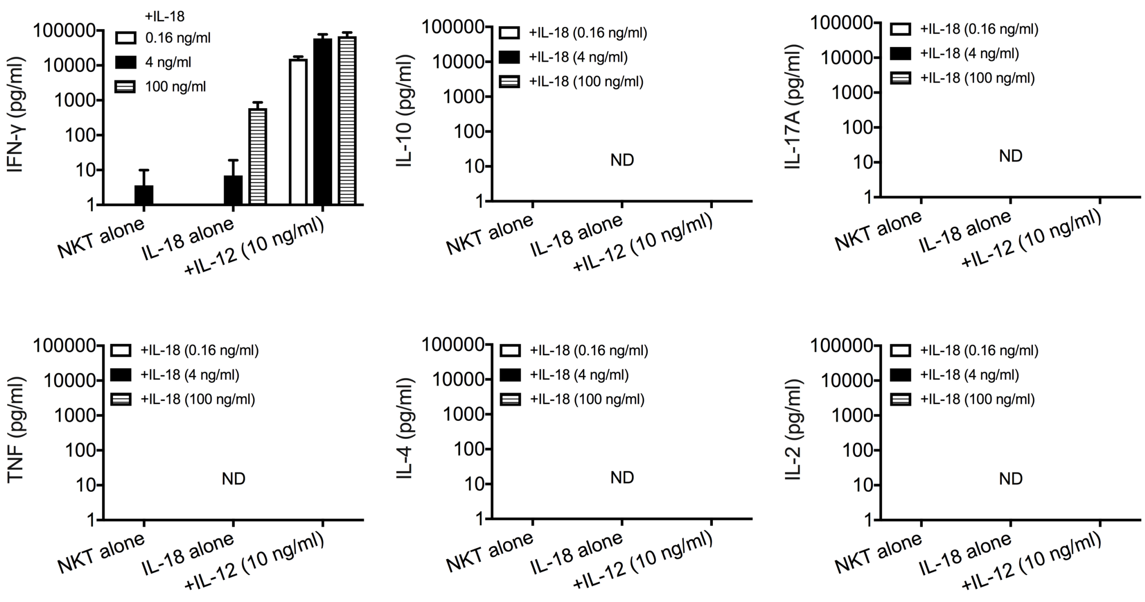
IFN-γ (pg/ml)
+IL-18
0.16 ng/ml
4 ng/ml
100 ng/ml
IL-10 (pg/ml)
IL-17A (pg/ml)
TNF (pg/ml)
IL-4 (pg/ml)
IL-2 (pg/ml)
+IL-18 (0.16 ng/ml)
+IL-18 (4 ng/ml)
+IL-18 (100 ng/ml)
100000
10000
1000
100
10
1
ND
NKT alone
IL-18 alone
+IL-12 (10 ng/ml)

Supplemental Figure 5. *Role of cytokines and cell bound signals in the activation of dNKT cells*. dNKT cells were MACS enriched (A) or FACS sorted (B, C) from spleens of 24αβ mice, and BMDC were derived from C57BL/6 mice or CD40$^{-/-}$ mice. (A) dNKT cells were cultured with BMDC in the presence of different TLR ligands. Concentration of IL-12p70 was measured in supernatants harvested at the indicated time points. Supernatants from triplicate wells were pooled and analyzed for IL-12p70 by ELISA. The results are from one of two similar experiments. (B) dNKT cells were co-cultured with C57BL/6 (wild-type, WT) or CD40$^{-/-}$ BMDC in the presence of different doses of IL-12 as indicated, and IFN-γ was measured by ELISA in culture supernatants after 24 hours (as in Figure 4B). Bars indicate mean and SD of triplicate cultures. Statistical comparisons are shown for NKT cells + WT-BMDC vs. NKT cells (*) and NKT cells + WT-BMDC vs. NKT cells + CD40$^{-/-}$BMDC (#) (ordinary two-way ANOVA). (C) dNKT cells were cultured in the presence of IL-12 and IL-18 as indicated in Figure 4D Flow cytometry based Th1/Th2/Th17 cytokine bead array analysis was performed with the culture supernatants from the experiments shown in Figure 4D. No other tested cytokines were detected besides IFN-γ, therefore only data from cultures with the highest amount of IL-12 are shown. Bars indicate mean and SD of three independent experiments. ND - not detectable; other tested cytokines were not detectable in these cultures, besides IFN-γ.

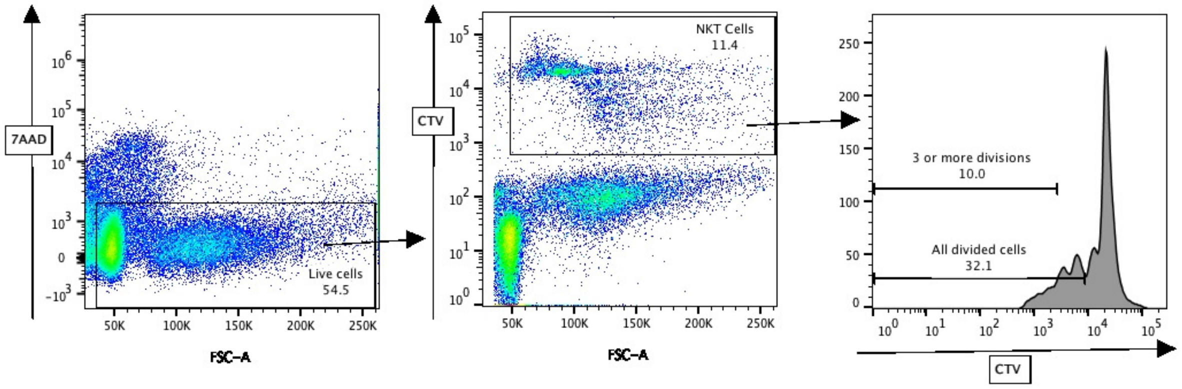

***Supplemental Figure 6**. Complete gating strategy for cell trace violet analysis of dNKT cell proliferation*. dNKT cells were FACS sorted from spleens of 24αβ mice, and BMDC were derived from C57BL/6 mice. Sorted dNKT cells were stained with cell trace violet (CTV) and co-cultured with BMDC+FSL1 (or other stimuli, see Figure 5). Cells were harvested after 60 hours and analysed for CTV dilution on BD-FACS LSR II after addition of 7AAD. All the CTV positive cells gated on total live cells (7AAD negative) were considered as dNKT cells and further represented in histograms for CTV fluorescence.

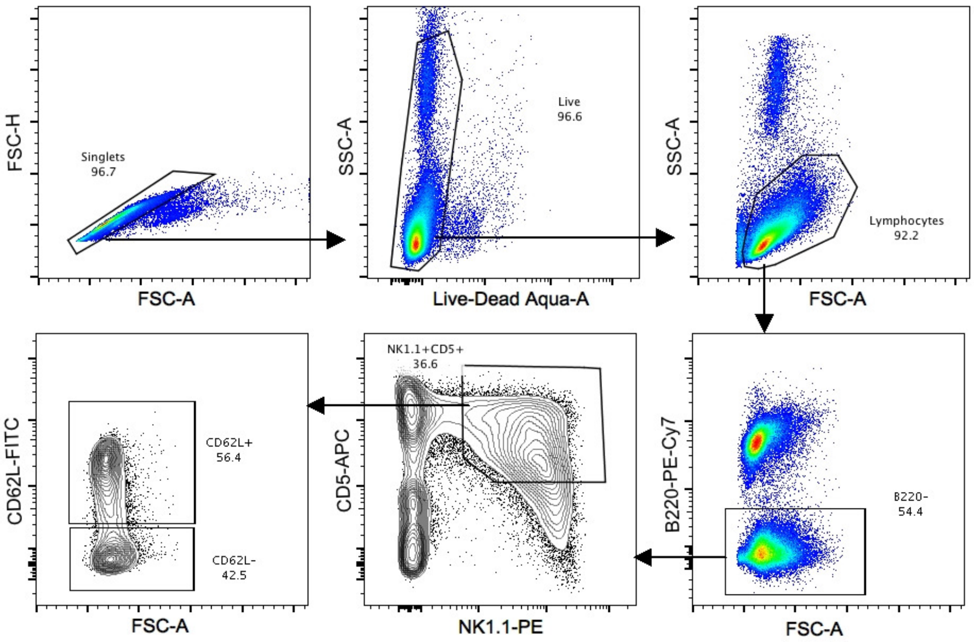

***Supplemental Figure 7**. Complete flow cytometry gating strategy to sort $CD62L^+$ and $CD62L^-$ dNKT cells*. dNKT cells were FACS sorted from spleens of 24αβ mice. $CD62L^+$ and $CD62L^-$ dNKT subsets were gated as shown and sorted from the $CD5^+NK1.1^+$ dNKT cell population and used for experiments.
