# Supplementary material for: Innate and adaptive stimulation of murine diverse NKT cells result in distinct cellular responses
Source: Eur J Immunol. 2018 Dec 3;49(3):443–53. doi: 10.1002/eji.201847647 (PMC6587840; doi:10.1002/eji.201847647)
Supplement: Supplementary file 1 — Peer review correspondence [file EJI-49-443-s001.pdf]

European Journal of Immunology

Innate and adaptive stimulation of murine diverse NKT cells result in distinct cellular responses

Prabhanshu Tripathi *, Saikiran Sedimbi, Avadhesh Kumar Singh, Linda Löfbom, Shohreh Issazadeh-Navikas, Siegfried Weiss, Irmgard Förster, Mikael C. I. Karlsson, Ulf Yrlid, Nadir Kadri and Susanna L. Cardell

Correspondence: Prof. Susanna Cardell, Box 435, Goteborg, SE-405 30

Tel: +46703769730

Review Timeline:	Submission date:	13-Apr-2018
	First Editorial decision:	24-May-2018
	Revision received:	24-Oct-2018
	Accepted:	31-Oct-2018

Handling Executive Committee member: Prof. Francesco Annunziato

Please note that the correspondence below does not include the standard editorial instructions regarding preparation and submission of revised manuscripts, only the scientific revisions requested and addressed.

First Editorial Decision
24-May-2018

Dear Prof. Cardell,

Manuscript ID eji.201847647 entitled "Innate and adaptive stimulation of diverse NKT cells result in distinct cellular responses" which you submitted to the European Journal of Immunology has been reviewed. The comments of the referees are included at the bottom of this letter. We are sorry for a delay in the peer review, we have received referees reports with some delay.

You will notice that our referees have raised serious concerns. You are strongly encouraged to address these concerns in great detail. In addition, the Executive Committee has requested that experiments in Figures 4 (A,B, E, F) and Figure 6 need to be repeated one more time, and statistics shown for pooled data from all experiments. A revised version of your manuscript that takes into account the comments of the referees will be reconsidered for publication. Should you disagree with any of the refereesâ€™ concerns, you should address this in your point-by-point response and provide solid scientific reasons for

why you will not make the requested changes.

You should also pay close attention to the editorial comments included below. **In particular, please edit your figure legends to follow Journal standards as outlined in the editorial comments. Please provide a figure with a full gating strategy and fluorochrome axis labels for all flow cytometry data. Failure to do this will result in delays in the re-review process.**

Please note that submitting a revision of your manuscript does not guarantee eventual acceptance, and that your revision will be re-reviewed by the referees before a decision is rendered.

If the revision of the paper is expected to take more than three months, please inform the editorial office. Revisions taking longer than six months may be assessed by new referees to ensure the relevance and timeliness of the data.

Once again, thank you for submitting your manuscript to European Journal of Immunology and we look forward to receiving your revision.

Yours sincerely,
Nadja Bakocevic

On behalf of
Prof. Francesco Annunziato

Dr. Nadja Bakocevic
Editorial Office
European Journal of Immunology
e-mail: ejied@wiley.com
www.eji-journal.eu

****

Reviewer: 1

Comments to the Author
The authors study the role of TLRL and IL12/15 and 18 mediated signals on the activation, proliferation and lymphokine secretion by Type 2 NKT cells using a TCRab double transgenic mouse system. For that purpose, after negative selection, enriched dNKT cells are stimulated in various settings. TLRL stimulating

INF-g secretion requires IL-12/18 and IL-15.
IFN-g secretion stimulated by IL-12 appears to require partly CD40 dependent interactions with cells.
IL-12 and IL-18 are strongly synergistic at inducing IFNg secretion. A CD62L based sorting is performed to show a slightly faster and wider lymphokine secretion for the CD62Llo subset. Besides using a series of KO APC, no real mechanistic study is performed regarding the role of membrane or soluble mediators expressed by the DCs.
One of the issue is whether the innate mediators and the lymphokines (IL-12/15/18) do not induce the upregulation of endogenous ligands binding to CD1d on the responding T cells themselves. To be completely demonstrative the authors may want to make mixed bone-marrow between CD1d-/- 24 abTCR together with a CD1d+ wt mouse to generate CD1d-/- 24 abTCR that could be used in functional assays. This may be beyond the scope of this paper.

The most interesting part is the IL12/18 interactions (Fig. 4). The authors studied the impact of adding a supra maximal (plateau is reached at 1 ng/ml in fig. 4A) dose of IL-12 (10 ng/ml) to various doses of IL-18. It would have been wiser to use several doses of IL-12. In fact, a complete double titration experiment would have been more informative. This should not be a problem as the source of dNKT cells is not limited. Other cytokines besides IFN-g should be tested in the supernatant as performed in fig. 6.
The significance of the CD62L sorting experiment in fig. 6 is unclear. Indeed, CD62L is not a good marker of memory as compared to CD44. In fact, in the original paper describing the 24 abTCR-double transgenic, most of the T cells that were selected in a CD1d dependent manner were CD44hi while half of them were CD62Lhi. The significance of this difference was not further studied in that paper. It is still not clear and the effects observed in fig. 6 are very small. In any case, considering that most of the T cells are CD44hi to qualify the CD62Lhi subset as naive is incorrect. Therefore, the biological significance of these findings is unclear.

Reviewer: 2

Comments to the Author
Tripathi et al. have conducted a comprehensive study to show that the innate (TCR ligand/DC) and adaptive (TCR/co-receptor) stimulation of dNKT cells resulted in distinct cellular responses. The conclusions the authors withdrew from performing an innate stimulation are convincing. However, under normal circumstances, TCR/co-receptor engagement also occurs in the presence of antigen-presenting cells (APCs), such as DCs. Therefore, the way the authors activated dNKT cells by anti-CD3/anti-CD28 in the culture plate, does not appear to represent the in vivo situation, which may lead to a misleading conclusion. In this regard, the authors need to address the outcome of the adaptive stimulation of dNKT cells in presence of APCs.

European Journal of Immunology

First Revision – authors' response

Manuscript eji.201847647

Point-by-point reply to editor's and reviewer's comments

We thank the Editor and reviewers for constructive comments. We have revised the manuscript as described below and hope that the revised manuscript is acceptable for publication.

Editor's comments

Comment: the Executive Committee has requested that experiments in Figures 4 (A,B, E, F) and Figure 6 need to be repeated one more time, and statistics shown for pooled data from all experiments

Reply: As requested, the experiments in Figure 4 (A, B, E and F) and Figure 6 have been repeated once. All panels have been changed, and the data shown are pooled from all experiments with statistics in all the panels, except for Fig 4B. For Fig 4B, the different experiments performed show the same result, however the absolute levels of IFN-□ produced varies between experiments making the SD in a Figure with pooled experiments very large, although the SD of triplicates in each experiment was low. Therefore we show one experiment in Fig 4B, and the other two experiments are shown in Supplemental Fig. 5B.

Comment: please edit your figure legends to follow Journal standards as outlined in the editorial comments

Reply: All figure legends have been edited to follow the Journal standards.

Comment: Please provide a figure with a full gating strategy and fluorochrome axis labels for all flow cytometry data.

Reply: Figures with full gating strategies have been provided for all flow cytometry data (see Supplemental Figures 1, 2, 3, 6 and 7).

Reviewer 1

Comment: One of the issue is whether the innate mediators and the lymphokines (IL-12/15/18) do not induce the upregulation of endogenous ligands binding to CD1d on the responding T cells themselves. To be completely demonstrative the authors may want to make mixed bone-marrow between CD1d-/- 24

European Journal of Immunology

abTCR together with a CD1d+ wt mouse to generate CD1d-/- 24 abTCR that could be used in functional assays. This may be beyond the scope of this paper.

Reply: Regarding a possible role of CD1d expressed on 24□□ T cells in the "innate" activation assays, we used CD1d blocking antibody when stimulating with DC and TLR ligands in Figure 1A. We demonstrate a partial role for CD1d when stimulating 24□□ dNKT cells with Fsl-1 + DC, but not when stimulating with DC and other TLR ligands. It is true that making the chimaera suggested by the reviewer would be an elegant way of confirming CD1d dependence/independence, however, as the reviewer also mentions, this would be a major undertaking and is beyond the scope of this study.

Comment: The most interesting part is the IL12/18 interactions (Fig. 4). The authors studied the impact of adding a supra maximal (plateau is reached at 1 ng/ml in fig. 4A) dose of IL-12 (10 ng/ml) to various doses of IL-18. It would have been wiser to use several doses of IL-12. In fact, a complete double titration experiment would have been more informative. Other cytokines besides IFN-g should be tested in the supernatant as performed in fig. 6.

Reply: We have performed a double titration experiments as suggested, and data are provided in new Figure 4D. We have tested other cytokines as requested, and could not detect other cytokines in the cultures. These results are mentioned on page 15, first paragraph, and shown in Supplemental Fig. 5C.

Comment: CD62L is not a good marker of memory as compared to CD44. In fact, in the original paper describing the 24 abTCRdouble transgenic, most of the T cells that were selected in a CD1d dependent manner were CD44hi while half of them were CD62Lhi. The significance of this difference was not further studied in that paper. It is still not clear and the effects observed in fig. 6 are very small. In any case, considering that most of the T cells are CD44hi to qualify the CD62Lhi subset as naive is incorrect. Therefore, the biological significance of these findings is unclear.

Reply: We agree that CD62L is not an good marker for memory. As noted, CD44, considered a marker of previous activation, is indeed expressed on the majority of TCR transgenic 24□□ dNKT cells. In Figure 6, we used CD62L to subdivide the dNKT cells, as we had previously demonstrated that this marker is differentially expressed among 24□□ dNKT cells. CD62L indicates a capacity to circulate through HEV in secondary lymphoid organs, while CD62L negative cells would not do this. We have changed the text in Results and mention this, and have omitted reference to "memory" and "naive" dNKT cells (page 16, lines 12-15). We have also changed the Abstract and Discussion (page 20 last paragraph, page 21 first paragraph and second last sentence on page) and describe the sorted dNKT cell subsets as CD62L+ and CD62L-. Figure 6 now shows pooled data from three experiments, and consistent differences between experiments have been pointed out in the Results on page 15.

Reviewer 2

Comment: under normal circumstances, TCR/co-receptor engagement also occurs in the presence of antigenpresenting cells (APCs), such as DCs. Therefore, the way the authors activated dNKT cells by anti-CD3/anti-CD28 in the culture plate, does not appear to represent the in vivo situation, which may lead to a misleading conclusion. In this regard, the authors need to address the outcome of the adaptive stimulation of dNKT cells in presence of APCs.

Reply: In this study, our primary goal was to investigate requirements for "innate" activation of dNKT cells. We also made a comparison of "innate" with "adaptive" stimulation. For this comparison, we selected a well established in vitro reductionist system for T cell activation regardless of TCR-specificity, anti-CD3 + anti-CD28, to mimic TCR stimulation. Clearly, in the presence of DC, additional signals could contribute to stimulation, however this would also add further complexity to the system. We therefore argue that in this study, anti-CD3/-CD28 is an acceptable (and widely used) system to provide basal TCR/co-stimulatory signals to mimic "adaptive" dNKT cell activation. It would be interesting to investigate further how additional DC-derived signals would influence TCR stimulation of dNKT cells, however, we feel that this is beyond the scope of the present study.

Second Editorial Decision

31-Oct-2018

Dear Prof. Cardell,

It is a pleasure to provisionally accept your manuscript entitled "Innate and adaptive stimulation of diverse NKT cells result in distinct cellular responses" for publication in the European Journal of Immunology. For final acceptance, please follow the instructions below and return the requested items as soon as possible as we cannot process your manuscript further until all items listed below are dealt with.

Please note that EJI articles are now published online a few days after final acceptance (see Accepted Articles: https://onlinelibrary.wiley.com/toc/15214141/0/ja). The files used for the Accepted Articles are the final files and information supplied by you in Manuscript Central. You should therefore check that all the information (including author names) is correct as changes will NOT be permitted until the proofs stage.

We look forward to hearing from you and thank you for submitting your manuscript to the European Journal of Immunology.

Yours sincerely,
Ivan Shevchenko

on behalf of
Prof. Francesco Annunziato

Dr. Ivan Shevchenko
Editorial Office
European Journal of Immunology
e-mail: ejied@wiley.com
www.eji-journal.eu
